# Nanogrid single-nucleus RNA sequencing reveals phenotypic diversity in breast cancer

**DOI:** 10.1038/s41467-017-00244-w

**Published:** 2017-08-09

**Authors:** Ruli Gao, Charissa Kim, Emi Sei, Theodoros Foukakis, Nicola Crosetto, Leong-Keat Chan, Maithreyan Srinivasan, Hong Zhang, Funda Meric-Bernstam, Nicholas Navin

**Affiliations:** 1 Department of Genetics, UT MD Anderson Cancer Center, Houston, TX 77030 USA; 2 Graduate School of Biological Sciences, UT MD Anderson Cancer Center, Houston, TX 77030 USA; 3 Department of Oncology-Pathology, Karolinska Institutet, 171 76 Stockholm, Sweden; 4 Department of Medical Biochemistry and Biophysics, Karolinska Institutet, 171 77 Stockholm, Sweden; 5 Wafergen Biosystems, Inc, 34700 Campus Drive, Fremont, CA 94555 USA; 6 Department of Pathology, UT MD Anderson Cancer Center, Houston, TX 77030 USA; 7 Department of Investigational Cancer Therapeutics, UT MD Anderson Cancer Center, Houston, TX 77030 USA

## Abstract

Single cell RNA sequencing has emerged as a powerful tool for resolving transcriptional diversity in tumors, but is limited by throughput, cost and the ability to process archival frozen tissue samples. Here we develop a high-throughput 3′ single-nucleus RNA sequencing approach that combines nanogrid technology, automated imaging, and cell selection to sequence up to ~1800 single nuclei in parallel. We compare the transcriptomes of 485 single nuclei to 424 single cells in a breast cancer cell line, which shows a high concordance (93.34%) in gene levels and abundance. We also analyze 416 nuclei from a frozen breast tumor sample and 380 nuclei from normal breast tissue. These data reveal heterogeneity in cancer cell phenotypes, including angiogenesis, proliferation, and stemness, and a minor subpopulation (19%) with many overexpressed cancer genes. Our studies demonstrate the utility of nanogrid single-nucleus RNA sequencing for studying the transcriptional programs of tumor nuclei in frozen archival tissue samples.

## Abstract (web summary)

Single cell RNA sequencing is a powerful tool for understanding cellular diversity but is limited by cost, throughput and sample preparation. Here the authors use nanogrid technology with integrated imaging to sequence thousands of cancer nuclei in parallel from fresh or frozen tissue.

## Introduction

The development of single cell sequencing technologies has revolutionized many diverse fields of biology over the last 5 years^1, 2^. Single cell RNA sequencing (RNA-seq) has provided new insights into cancer progression by resolving complex cell types^3–5^, developmental hierarchies^3, 4, 6^, and phenotypic plasticity^7, 8^. However, initial methods were limited by low-throughput, high costs and extensive technical errors, which inhibited their broad application in cancer research^9–11^. Recent technological innovations using microwells^12–14^ and microdroplet encapsulation^15, 16^ have increased the throughput of single cell RNA-seq to thousands of cells and greatly reduced associated costs. However, high-throughput methods do not enable imaging or selection of single cells, leading to high doublet error rates and the inclusion of many unwanted cells, such as dead cells^11^. Furthermore, the ability to sequence RNA in nuclei instead of whole cells on these platforms has not been demonstrated.

A second major challenge for single cell RNA-seq in cancer research is that most methods require fresh tissue to be dissociated into single cell suspensions for analysis^17^. This is logistically challenging and problematic in cancer research, since most archival tissue samples have previously been flash frozen and stored in cryobanks, a process that ruptures the cell membranes. However, previous work has shown that nuclear membranes remain intact during freeze-thaw cycles, and that single nuclei can be isolated from frozen tissues^18^ that allow nuclear suspension preparation^19–21^ and construction of cDNA libraries while avoiding the use of proteases to dissociate whole cells^18^. Neuroscientists have also shown that RNA-seq of single nuclei is feasible and highly representative of transcriptional profiles from cells, when fresh tissues are dissociated^18, 22–24^ and even when postmortem brain stored long term at −80 °C is used^18^. This is in contrast to whole brain cells, where the use of proteases for whole-cell dissociation has been shown to activate the crucial immediate early genes^25^. However, to date, no one has investigated the transcriptional profiles of single tumor nuclei, to determine if they are representative of whole tumor cells.

To address these limitations, we developed a nanogrid platform and microfluidic depositing system that enables imaging, selection, and sequencing of thousands of single cells or nuclei in parallel. We applied this nanogrid single-nucleus RNA-seq (SNRS) system to compare the transcriptional profiles of cancer cells and nuclei in cell lines and further applied this method to study phenotypic diversity and subpopulations in a frozen tumor sample from a triple-negative breast cancer (TNBC) patient.

## Results

### Concordance of bulk nuclei and cells from cell lines

**Fig. 1:**
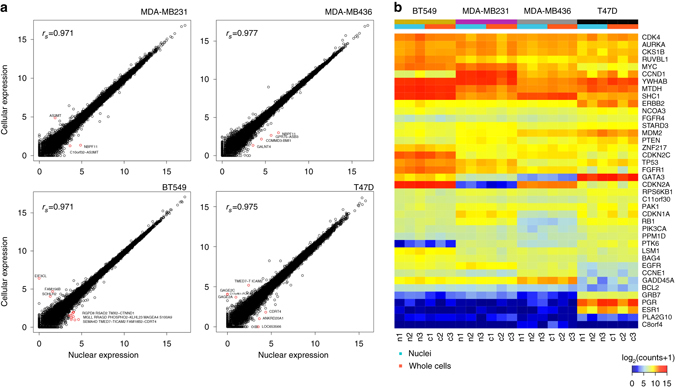
Bulk nuclear and cellular expression profiles from cell lines. **a** Scatter plots of gene expression [log_2_(counts + 1)] from bulk cellular and nuclear fractions isolated from four breast cancer cell lines. Significantly differentially expressed genes are highlighted in *red* and Spearman correlations are indicated. **b** Gene expression heat map of 40 breast cancer genes identified in TCGA across three biological replicates of bulk cell and nuclei fractions from the four breast cancer cell lines

Prior to single cell analysis, we investigated whether the transcriptional profiles of bulk cells and nuclear fractions are concordant in breast cancer cell lines. We performed RNA-seq of nuclear and cellular fractions isolated from millions of cells from four breast cancer cell lines, including three triple-negative subtypes (BT549, MDA-MB231, and MDA-MB-436) and an ER+/PR+ subtype (T47D). Nuclear fractions were purified from cellular suspensions using a detergent to lyse the plasma membrane, followed by three rounds of purification to eliminate residual cytoplasmic RNA (Online Methods). The nuclear suspensions were imaged in bright field and fluorescence using DAPI to ensure that cellular membranes and cytoplasm was no longer present (Supplementary Fig. 1). RNA-seq was performed on the nuclear and cellular fractions from each cell line at 20 million reads/sample, resulting in 50% of the reads mapping to the CDS regions and 15–16K gene coverage for each cell line. Correlations in gene expression levels between the nuclear and cellular suspensions were very high (*r*_s_ > 0.9), with only 3–38 discordant genes in each sample (Fig. 1a). In total, BT549 showed 13/15,699 discordant genes, while MDA-MB231 showed only 3/15,592 discordant genes, MDA-MB-436 showed 4/16,479 discordant genes, and T47D showed 6/16,314 discordant genes between cells and nuclei. Importantly, most genes had differences in expression levels, rather than having a complete absence of expression in the cells or nuclei. However, *EIF3CL* was only detected in BT549 whole cells and was not detected in the nuclei. We speculate that this mRNA is the result of rapid transportation between cellular compartments, since the sequencing depth was sufficiently high in both samples to rule out false negatives. Other genes, such as *NBPF11*, were found to have 2.1-fold-increased expression in the nuclei of MDA-MB436 and 3.5-fold-increased expression in the nuclei of MDA-MB231. Notably, six differentially expressed genes were read-through transcripts (Fig. 1a). We also examined the gene expression levels for a targeted set 40 breast cancer genes that were previously reported to be frequently deregulated in a study in The Cancer Genome Atlas project^26^. These data show only minor variations between cells and nuclei that were not statistically significant (false discovery rate (FDR)-adjusted *p* value ≥0.05 or |log_2_(fold change)| < 1) across three biological replicates in each of the four cell lines (Fig. 1b). Collectively, these experiments suggested that the transcriptional profiles of bulk nuclear RNA are highly representative of cellular RNA in breast cancer cell lines.

### Nanogrid SNRS

**Fig. 2:**
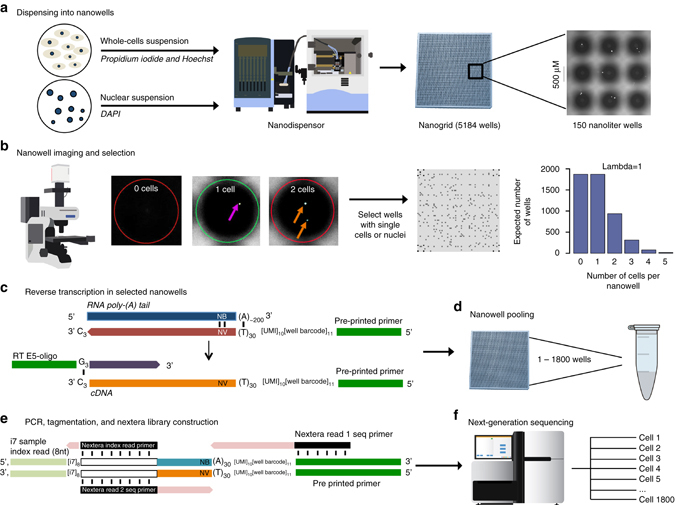
Overview of the nanogrid single-nucleus sequencing method. **a** Nuclear or cellular suspensions are prepared and stained with DAPI (nuclei) or Hoechst and propidium iodide (cells) for nanodispensing into the 5184 wells in the nanogrid. Each nanowell is 400–600 μm, and the well depth range is 950 μm–2.2 mm. The 500 μm *scale bar* indicates the well diameter. **b** The nanowells are imaged using automated scanning fluorescent microscopy and ~1800 wells containing single nuclei or cells are selected, while nanowells containing multiple cells, no cells or dead cells are excluded. **c** In the selected nanowells, the nanodispensor deposits lysis buffer and WTA reagents to perform reverse transcription of mRNA to cDNA using SCRB-Seq chemistry. This process also adds a UMI, well barcode and P5 adapter sequence to the (A)_n_ tail. N represents A, C, G, or T; B represents C, G, or T; and V represents A, C, or G. **d** The barcoded cDNA with adapter sequences is pooled into a single reaction. **e** Nextera tagmentation is performed followed by PCR amplification to generate sequencing libraries with Illumina I7 indexes. **f** Next-generation sequencing is performed on the pooled libraries, after which the individual cell data is demultiplexed using the well barcodes

We developed a high-throughput nanogrid SNRS approach by incorporating the ICELL8 system (Wafergen, Inc.) (Fig. 2, Online Methods). The nanogrid system consists of three main components: (1) an alloy nanogrid with 5184 nanowells, (2) a nanodispensing system, and (3) an automated imaging system. First, single cell or single-nucleus suspensions are prepared from cell lines or frozen tissues and stained with propidium iodide (PI) and Hoechst (cells) or stained with DAPI (nuclei). The suspensions are then diluted to one cell or nucleus/50 nL and dispensed into the nanogrid resulting in a Poisson distribution of cell occupancies across the 5184 nanowells with ~1800 wells that are expected to have single cells or nuclei (Fig. 2a, and Supplementary Fig. 2). Each well in the nanogrid has a unique 11 bp well barcode (WBC) that is preprinted in the chip that also contains an oligo dT sequence, a 10 bp unique molecular identifier (UMI) and a P5 Illumina adapter sequence. After the cells or nuclei are dispensed, automated imaging is performed within 10 min using a fluorescent microscope with a robotic stage to image all 5184 wells (Fig. 2b). The software automatically identifies nanowells containing single cells or nuclei, and excludes wells with doublets or no cells. The user can then choose to manually select a subset of the prioritized cells based on the morphology parameters, PI, Hoechst, or DAPI staining. Only wells with live cells (PI−, Hoechst+) or intact nuclei (DAPI+) are selected for depositing reagents for lysis and whole-transcriptome amplification (WTA) (Fig. 2b). The WTA reaction is performed using SCRB-Seq^27^, which uses template switching to select polyadenylated RNA and incorporate a P5 adapter sequence along with the WBC and UMI into the 3′ end of each RNA molecule (Fig. 2c). A second adapter is added to the 5′ end (RT-E5OLIGO) by template switching for second-strand synthesis and subsequent PCR enrichment. The nanogrid is then inverted and all of the barcoded cell libraries are pooled together into a single reaction for PCR amplification followed by QC for size distributions (Fig. 2d). The cDNA is then used to generate Illumina sequencing libraries with one-sided tagmentation and PCR amplification (Fig. 2e). A detailed figure of the steps involved in constructing barcoded libraries from nanowell adapters is provided in Supplementary Fig. 2 and the Poisson distribution of single cells and nuclei in nanowells is shown in Supplementary Fig. 3. The pooled libraries are then sequenced on the Illumina system, and individual FASTQ files are demultiplexed using the WBC identifiers for downstream data processing and analysis (Fig. 2f and Supplementary Fig. 4).

### Single nuclei and cell concordance in a cancer cell line

**Fig. 3:**
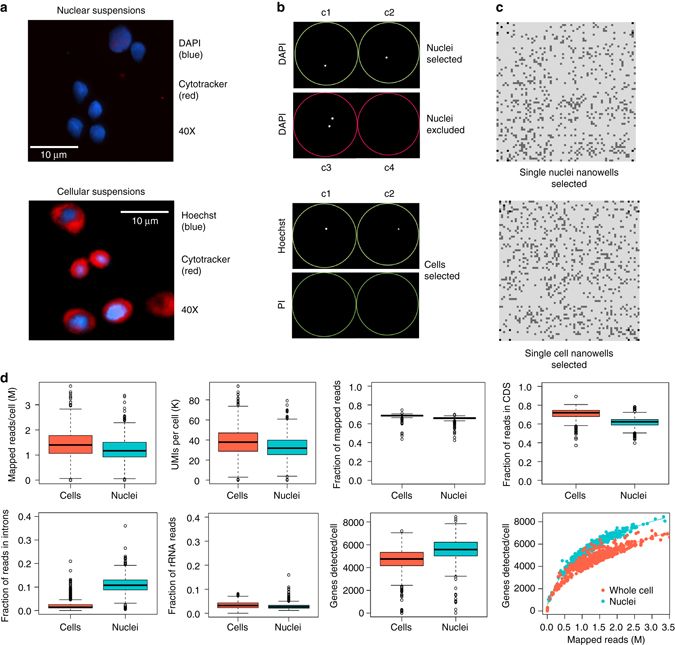
Imaging and data metrics of single SK-BR-3 nuclei and cells. **a** Nuclear suspensions were stained with DAPI (*blue*) and cytotracker (*red*), while cellular suspensions were stained with Hoechst (*blue*) and cytotracker (*red*) for fluorescent microscopic imaging at ×40 magnification to visualize the nucleus and cytoplasm. The 100 μm *scale bar* indicates the cell size as a reference. **b** Nanodispensed nuclei and cells were imaged in nanowells using DAPI, or Hoechst and PI **c** Nanogrid maps of cells or nuclei that were selected for RNA-seq. **d** Sequence data metrics were calculated for single nuclei and single cells from the SK-BR-3 breast cancer cell line, where the *boxes* indicated the 75% interquartile range and *whiskers* showed the default values of the *boxplot*, with *N* = 485 for single nuclei and *N* = 424 for whole cells

To determine if the transcriptional profiles of single nuclei were representative of whole cells, we applied nanogrid SNRS to isolate and sequence nuclei and cells from an isogenic Her2+ breast cancer cell line (SK-BR-3) (Fig. 3). Nuclear and cellular suspensions were prepared and stained with a nuclear stain (Hoechst) and cytoplasmic stain (cytotracker) to confirm that the cytoplasmic membrane was no longer intact in the nuclear suspensions (Fig. 3a). We subsequently stained the nuclear suspensions with DAPI and the cellular suspensions with PI and Hoechst, and dispensed single cells into the nanogrid for automated imaging (Fig. 3b). In total, we selected 525 single nuclei and 525 live cells in the nanogrids for sequencing (Fig. 3c).

To understand global expression differences in the data, we computed a number of metrics for the single cell and nuclei data sets (Fig. 3d). On average, we sequenced 1.3 million reads/nucleus or cell, achieving a coverage of 4600–5500 genes and average unique molecular identifier (UMI) count of 34,690 (±12,609 SD) for cells and 34,540 (±10,570 SD) for nuclei. Single cells or nuclei with poor metrics were filtered, resulting in final data sets of 485 nuclei and 424 whole cells (Online Methods). Although the difference in variance for most distributions was determined to be significant by Kolmogorov-Smirnov test (*P* < 0.05) due to large numbers of nuclei in each distribution, most metrics, including mapped read fractions, 5′ UTR tags, 3′ UTR tags, rRNA reads, and gene coverage showed very low percent changes (2–5%) between nuclei and cells. However, we did observe an 8.5% increase in intron tags and 9.2% decrease in CDS tags in the single nuclei data, which is consistent with the dogma that nuclei contain unprocessed pre-mRNA that have not yet undergone splicing and export from the nucleus^28, 29^. To compare our data to another platform, we performed Drop-Seq analysis of SK-BR-3 cells and compared the sequencing metrics to downsampled nanogrid RNA data (Supplementary Table 4). These data revealed similar per cell gene-detection rates, but higher UMI counts/cell on the nanogrid platform.

**Fig. 4:**
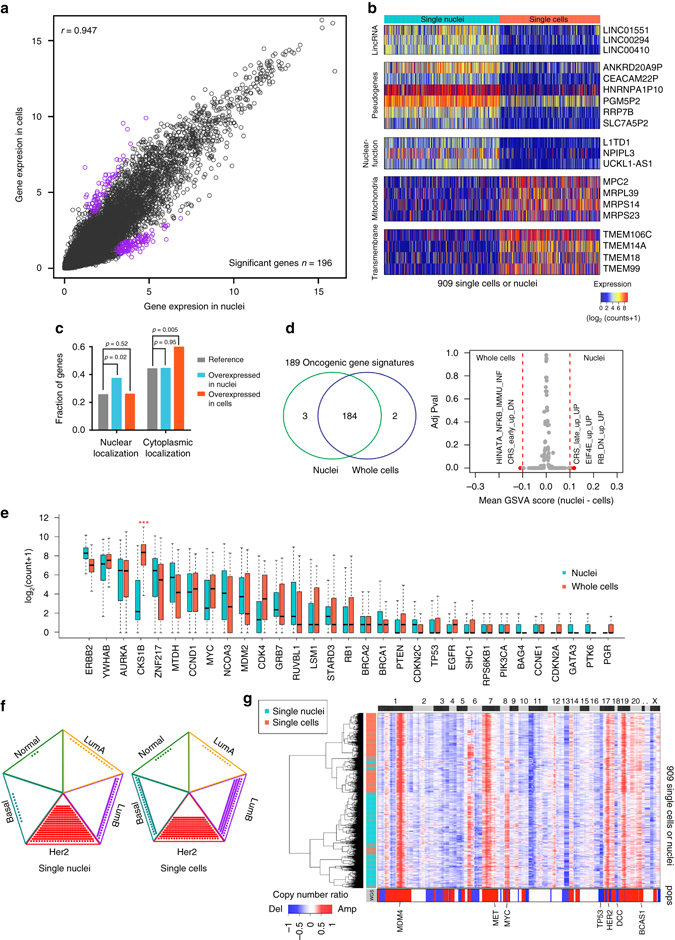
RNA-seq of single nuclei and cells in SK-BR-3. **a** *Scatter plot* of average gene expression [log_2_(count + 1)] of 485 single nuclei and 424 single cells, with 196 significantly differential genes labeled in *purple* and Spearman’s correlation values indicated. **b** *Heat map* of selected differentially expressed genes that are LincRNAs, pseudogenes, nuclear-function genes, mitochondria or transmembrane. **c** Protein and RNA localization enrichment analysis comparing genes overexpressed in the nucleus or cells. **d** *Venn diagram* and *volcano plot* of GSVA scores for 189 oncogenic gene sets expressed in nuclei or cells. **e** *Boxplots* for 40 TCGA breast cancer genes grouped by nuclei or cells, with a *star* indicating significant differential expression in the CKS1B gene (SCDE model, FDR-adjusted *p* value <0.05). **f** Breast cancer subtypes of single cells or nuclei predicted with the PAM50 gene signature. The frequency concordance was determined as non-significant by the Wilcoxon-matched pairs signed-rank test, where *p* value >0.4. **g** Clustered *heat map* of single cell or nuclei copy number profiles calculated from RNA data, compared to whole-genome-sequencing data labeled as pop. Breast cancer genes are annotated on the WGS track

We next investigated whether any biological differences could be detected between nuclear and cellular transcriptomes. Our data suggest that the overall expression level and abundance of genes are very similar (*r*_s_ = 0.95) between nuclei and whole cells, consistent with the bulk experiments (Fig. 4a). Analysis of gene expression variance showed a bell-shaped curve, high correlation (*ρ* = 0.451, Spearman correlation) of gene expression values between all cells, and gene dropouts at lower expression levels, justifying for the use of the zero-inflated negative binomial single cell differential expression (SCDE) statistical model (Supplementary Fig. 5). In total, we detected only 6.66% (196/2942) genes that were significantly differentially expressed using SCDE^30^ (FDR-adjusted *p* values <0.05 and |log_2_(fold change)|> = 1). Gene ontology and pathway analysis of these genes showed higher levels of LincRNAs, pseudogenes, and nuclear-function genes in the nucleus compared to cells, while conversely the nuclei showed low levels of mitochondrial and transmembrane genes, which showed higher levels in the cells (Fig. 4b and Supplementary Tables 1 and 2). We also investigated whether known RNA or protein localization in specific cellular compartments showed any correlation with whether RNA was expressed at high levels in the nuclei versus whole cells (Fig. 4c). These data show that proteins and RNA that localize to the nucleus are significantly elevated in nuclei, whereas proteins and RNA that localize to the cytoplasm are higher in cells.

We further investigated whether any cancer genes or pathways were differentially expressed in the nuclei and whole cells. We performed gene set variation analysis (GSVA) analysis^31^ using 189 oncogenic gene set signatures (MSiDB v5.2)^32^, which showed that most signatures (97.35%) were highly concordant, but that 5/189 gene sets were differentially expressed (Fig. 4d and Supplementary Table 3). The differentially expressed gene signatures include three pathways that were upregulated in the nucleus (*EIF4E* translation and nuclear export factors, *RB* cell cycle signaling, and CSR serum starvation response up-regulated) and two pathways that were upregulated in the whole cells (*NFKB* inflammation and CSR serum starvation response up-regulated). We also examined the expression levels of 40 breast cancer genes that are frequently deregulated in TCGA^26^ (Fig. 4e). Only one gene, CDC28 protein kinase regulatory subunit 1B (*CKS1B*), was found to be significantly lower (2.4-fold) in the nuclei compared to the cells. We also applied the PAM50 gene signature^33^ to classify single cells into the five major clinical breast cancer expression subtypes (normal-like, luminal A, luminal B, human epidermal growth factor receptor 2 (Her2) or basal-like). The relative frequency shifted slightly in each group, however, it was not significant by concordance test (*p* value >0.4). These data showed that most of the single cells and nuclei were classified as Her2 positive, consistent with the report that SK-BR-3 is a Her2-positive cell line by immunohistochemistry^34^. However, we also found that a subset of the single cells were classified as luminal B (10.7, 23.6%), basal-like (1.8, 5.9%), normal-like (0.4, 1.4%), or luminal A (2.4, 3.3%) in both the nuclei and cells (frequency concordance *P* > 0.4), suggesting that the population of cells represented a composite mixture of molecular subtypes (Fig. 4f).

We used the single cell gene expression data to calculate genomic copy number profiles at ~1 megabase resolution (Online Methods). A similar approach was previously applied to single cell RNA-seq data from glioblastoma patients^3^. In our data, we found that the copy number profiles of nuclei and whole cells were highly concordant (Pearson’s *r* = 0.91) and identified several large-scale amplifications, including 1q (*MDM4*), 7p, 8q (*MYC*), 17q (*HER2*), 19 and 20 (*BCAS1*) and deletions of 6q, 11p, 13, 17p (*TP53*), 18 (*DCC*) and Xp (Fig. 4g). However, neither the single cell nor nuclei copy number profiles could accurately resolve the smaller (<10 mb) chromosome deletions and amplifications and did not detect the *MET* amplification on chromosome 7q. The correlation between the single cell or nuclei profiles and the whole-genome sequencing was *r* = 0.38 (Pearson correlation). Collectively, these data suggest that transcriptome profiles of nuclei are highly representative of whole cells, and can be used to study many cancer genes and signaling pathways.

### Phenotypic diversity of single nuclei from a breast tumor

**Fig. 5:**
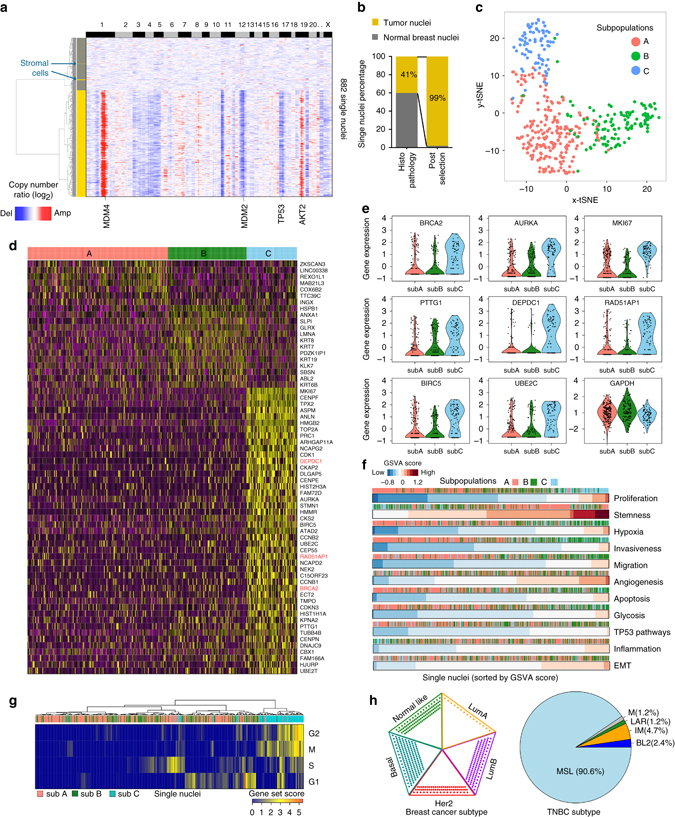
Single-nuclei sequencing of a TNBC. **a** Clustered *heat map* of single nuclei RNA copy number profiles isolated from tumor tissue or normal breast tissue. *Green arrows* indicate five stromal cells from the tumor with diploid copy number profiles. *Bar-plot* shows increase in tumor purity after image selection of larger nuclei. **b** Enrichment of tumor cells using nanogrid SNRS from 41 to 99%. **c** t-SNE projection of single nuclei in high-dimensional space with SNN clustering of three subpopulations **a**, **b**, **c** indicated by *color*. **d** Clustered *heat map* of single nuclei RNA expression profiles using differentially expressed genes. Cancer genes are highlighted in *red* and the three major clusters are indicated by *color*. **e** *Violin plots* of single cells expression levels of individual genes across the three tumor subpopulations. **f** GSVA scores for a subset of oncogenic phenotypes with single nuclei sorted by each row independently with *header bars* indicating their identities of the predicted subpopulations. **g** Clustered *heat map* of cell cycle signature scores for single nuclei with subpopulation indicated in the header. **h** Subtype signature classifications for single tumor nuclei predicted by PAM50 for the five breast cancer subtypes (*left*) and for the six TNBC subtypes (*right*)

We applied nanogrid SNRS to study tumor subpopulations and transcriptional diversity in a triple-negative (ER−, PR−, and HER2−) breast tumor that was cryopreserved for 2 years. Nuclear suspensions were generated from the frozen tumor and 502 nuclei were sequenced using cDNA obtained with the nanogrid platform. In total, we identified 1421 wells with single nuclei during the imaging step and selected 502 wells with larger nuclei (>8 microns) to increase the tumor purity and avoid sequencing normal stromal cells. The tumor purity was estimated by histopathology to be 41%. The data metrics resulted in an average of 975,097 reads, 14,886 UMI counts, and 3619 genes/nucleus (Supplementary Fig. 6). We also used nanogrid SNRS to sequence 380 nuclei from a normal breast tissue sample. To distinguish between tumor and stromal cells, we calculated the genomic copy number profiles of each nucleus and clustered the data together with the normal breast tissue nuclei that showed diploid copy number profiles (Fig. 5a). In our previous work, we have shown that most tumor cells in breast cancer are aneuploid, while most normal cells have diploid profiles with no evidence of somatic mutations^20, 21^. The hierarchical clustering of the copy number data identified 5/502 cells with normal diploid copy number profiles representing stromal cells (Fig. 5a, *green arrows*) and 497 cells with aneuploid tumor profiles. The aneuploid tumor cells had amplifications in chromosomes 1q (*MDM4*), 19q (*AKT2*), and deletions on chromosome 1p, 3, 4, 11p, 12q (*MDM2*), 17p (*TP53*), 19q, 22. These data suggest that the purity of the tumor cells was increased from 41 to 99% using the automated imaging and selection of nanogrid wells with larger nuclei (Fig. 5b).

We first applied MAST^35, 36^ to identify differentially expressed cancer genes (FDR-adjusted *p* value <0.05 and [log_2_ (fold change)] ≥ 1) between single tumor cells (*N* = 497), stromal cells (*N* = 5), and normal breast cells (*N* = 240). This analysis identified 30/229 known cancer genes (T200 targeted platform) and 11/40 TCGA breast cancer genes that were differentially expressed relative to the normal breast cells, including *KRAS*, *GATA3*, *CCND1*, *CDH1*, *GNAS*, and several others. Most of these genes were expressed across all tumor cells, however, a few cancer genes, including AURKA and TOP2A, were restricted to a minor subpopulation (Supplementary Fig. 7).

To further understand phenotypic variability within the tumor cells, we focused on the 416 aneuploid tumor cells that past our QC filters (Online Methods) and performed principle component analysis (PCA) linear dimension reduction to identify the top variable genes from the first five principal components (49.34% variance explained) for unsupervised clustering using shared nearest neighbor (SNN) modularity optimization^37^ (Fig. 5c) and t-distribution stochastic neighbor embedding (t-SNE)^38^, which was implemented using the SEURAT package^15^ (Fig. 5c-e). This analysis identified three subpopulations of tumor cells (clusters A, B, and C). Subpopulation A consisted of 217 nuclei (52.2%) and showed overexpression of seven genes compared to other tumor subpopulations, which did not include any known cancer genes. Subpopulation B consisted of 121 cells (29.1%) and showed overexpression of 13 genes, including seven cancer genes (*HSPB1*, *ANXA1*, *SLPI*, *KRT8*, *KRT19*, *KLK7*, and *ABL2*) and several Keratin genes (*KRT8*, *KRT7*, *KRT19*, and *KRT6B*). Subpopulation C was the rarest subpopulation (18.8%), consisting of 78 cells, but had the highest number of cancer genes (*N* = 13) that were overexpressed (*MKI67*, *TPX2*, *TOP2A*, *PRC1*, *CDK1*, *AURKA*, *CKS2*, *BIRC5*, *DEPDC1 UBE2C*, *NEK2*, *BRCA2*, and *RAD51AP1*). Several genes that were differentially expressed in subpopulation C are involved in DNA damage repair (*BRCA2*, *RAD51AP1*, and *HMGB2*), apoptosis (BIRC5, *DEPDC1*) and mitosis or cell cycle regulation, including high expression of Ki-67 (*MKI67*), a marker of cell proliferation. To determine if the expression of the 13 cancer genes were truly restricted to the minor C subpopulation, we plotted the individual gene expression levels of single cells using Violin plots (Fig. 5e). These data confirmed that most of the cancer genes were highly elevated in subpopulation C, and had low expression in the A and B subpopulations.

Due to the high levels of Ki-67 in subpopulation C, we performed gene signatures analysis for the cell cycle stages (G1, S, M, and G2) in all of the tumor nuclei (Fig. 5g). These data showed that many of the subpopulation C tumor cells were in the G2 or M phase of the cell cycle, suggesting an actively proliferating subpopulation, while many cells from subpopulation A and B were in the G1 phase or G0 (absence of scores). Our data suggest that while subpopulation C was the minor subpopulation in the tumor mass, it also had the most malignant cancer phenotypes. The nuclei in subpopulation C are likely to be in the G2 or M phase, because the nuclear membrane does not break down until prometaphase of mitosis and is reformed during telophase. Therefore, there are still some nuclei in prophase, prometaphase, telophase, and cytokinesis that are mitotic. Interestingly, the imaging data of nuclear size estimated from nuclear signal intensity showed that cells in the G2 or M phase had significantly larger sizes compared to other stages of the cell cycle (Supplementary Fig. 8), which is consistent with previous data on the nuclear diameters of G2/M cells^39, 40^. Our analysis of the cell cycle data estimate that 18.75% of the cells were in the G2/M stage of the cell cycle, suggesting that we did not bias strongly against other cell cycle stages by the selection of larger nuclei by DAPI staining during the nanowell imaging steps.

We further investigated the diversity of the classical breast cancer subtypes and oncogenic gene signatures at single cell resolution. GSVA analysis identified variation in the gene signatures for a number of cancer phenotypes, including stemness, proliferation, and angiogenesis (Fig. 5f). Consistent with our previous analysis, we found that subpopulation C was enriched for cell proliferation, while subpopulation A showed low scores for proliferation. The GSVA data further showed that subpopulation B had higher scores for hypoxia, invasiveness, migration, apoptosis, and glycolosis. Next, we used the PAM50 gene signature^33^ to investigate the diversity of the five major breast cancer subtypes, and found that most cells were of the basal-like (156/416) subtype, which is expected since this subtype is commonly associated with TNBC patients^33^. However, we also identified a significant fraction of single tumor nuclei that were Her2 positive (15.6%), luminal A (3.4%), luminal B (26.4%), and normal like (17.1%), suggesting that the tumor was a mixture of different subtypes (Fig. 5h). Studies have also shown that TNBC patients can be further classified into six additional subtypes based on gene expression signatures: mesenchymal (M), mesenchymal stem-like (MSL), luminal androgen receptor (LAR), immunomodulatory (IM), basal-like 1 (BL1) and basal-like 2 (BL2)^34^. We applied the TNBC subtype signature to the tumor nuclei predicted by PAM50 to be basal-like, which showed that most nuclei were classified as MSL (90.6%), but that a few cells were IM (4%) or belonged to the other TNBC subclasses (Fig. 5h). These data suggest that single tumor nuclei show diverse cancer phenotypes and subtype classifications within a single patient’s tumor.

## Discussion

In this study we report a nanogrid SNRS technology that performs high-throughput single-nuclei imaging, selection, and sequencing in an integrated platform. Nanogrid sequencing has several technical advantages over existing high-throughput single cell RNA-seq methods that use microwells^12–14^ or microdroplet encapsulation^15, 16^. Our system enables automated imaging of the 5184 wells that contain single cells or nuclei with fluorescent channels, followed by selection of specific nanowells for nanodepositing of WTA reagents. Imaging and selection of single cells is not technically feasible using microdroplet or microwell methods^15, 16^. With nanogrid imaging, we can reduce cell doublets and exclude dead cells by imaging. Another technical study using the nanowell platform estimated the doublet error rate to be 2.4% by human-mouse mixing experiments^41^. In addition, by staining nuclei with DAPI, our approach can select larger nuclei, and thereby increase the purity of the tumor cells to 99%. This addresses a major issue in standard RNA-seq studies of tumor tissues in which many normal stromal cells often affect gene signature analysis. While a previous study has combined robotic micromanipulation and imaging^42^ to perform low-throughput single cell RNA-seq (about 10 min/cell) and individual single cell library construction, the nanogrid system completes imaging of all 5184 wells in only 10 min and requires only a single sequencing library to be constructed for sequencing analysis. This greatly reduces the cost to about $2.20/cell, with the cost per 1800 cell library at $1.10/cell and the sequencing cost at $1.10/cell for achieving 220K reads/cell on a HiSeq4000 system (Illumina). Another study developed a nanowell system that uses barcoded mRNA capture beads to create a portable, low-cost system called Seq-Well^43^. While very cost effective and high throughput, this system does not allow selection of imaged nanowells, which is an advantage of our platform.

We applied the nanogrid SNRS to study the transcriptional differences of cells and nuclei in a breast cancer cell line, which showed a high concordance in transcript abundance and expression levels. These data challenge the long-standing paradigm that nuclear transcriptomes are not representative of whole cells. However, our data are consistent with recent studies in neural cell types that have reported a high concordance between the transcriptional profiles of nuclei and whole cells^18, 22–24^. We further applied nanogrid SNRS to study a frozen tumor sample from a TNBC patient that was cryopreserved for about 2 years. On a parenthetical note, we do not expect that length of freezing will have a major effect on the stability of nuclear RNA, however, multiple freeze-thaw cycles may lead to RNA degradation. In the TNBC tumor data, we identified a minor (19%) subpopulation of tumor cells that were highly proliferative and overexpressed many cancer genes. Our data also showed phenotypic heterogeneity in stemness, angiogenesis, and proliferation, as well as the co-existence of multiple breast cancer subtypes in single cells from an individual tumor. These data are consistent with a recent single cell RNA-seq study in glioblastoma that showed variation in EMT and the co-existence of many clinical subtypes within the same patient’s tumors. This striking amount of pre-existing phenotypic variation may explain why TNBC patients evolve rapid resistance to neoadjuvant chemotherapy^44–46^.

Although the majority of nuclear and cytoplasmic genes were concordant, we did identify a few differences in LincRNAs, pseudogenes, mitochondrial genes, and nuclear-function genes. These data are consistent with previous data showing that pseudogenes and LincRNAs are transcribed and preferentially located in the nucleus over the cytoplasm^47, 48^. Our data also show that mitochondrial genes are not expressed at high levels in the nucleus, which is consistent with the localization of the mitochondria in the cytoplasm^49^. We also found an increased abundance of intronic sequences in the nucleus, which is expected based on our knowledge of alternative splicing of pre-mRNAs in the nucleus^28, 29^. Importantly, our data suggest that these gene expression differences did not have a major influence on the measurements of most cancer genes and signaling pathways.

In closing, the SNRS nanogrid system opens up new avenues of investigation into the analysis of single nuclei transcriptomes from frozen tissue sections. In addition to imaging live and dead cells, or nuclear size as estimated from intensity, the nanogrid imaging approach is flexible and can be applied broadly to identify cell types of interest based on fluorescent markers. We expect that the SNRS nanogrid approach will benefit not only cancer research, as demonstrated in this study, but will also benefit many diverse fields of biomedical research, where the analysis of single nuclei from frozen tissue samples can provide new insights into human diseases.

## Methods

### Breast cancer tissue samples and cell line

The SK-BR-3 breast cancer cell was obtained from Characterized Cell Line Core (CCLC) Facility at the University of Texas MD Anderson Cancer Center, Houston, TX. The cell line was tested and found to be mycoplasma-negative and authenticated with the short tandem repeat method by the CCLC facility. A frozen tumor sample was obtained from an invasive ductal carcinoma patient in collaboration with Dr Hong Zhang and Dr Funda Meric-Bernstam at MD Anderson Cancer Center. The study was approved by the internal review board at the University of Texas MD Anderson Cancer Center and the patient consented to the study. The triple-negative status of this tumor sample was determined by immunohistochemistry for estrogen receptor (<1%) and progesterone receptor (<1%), and fluorescence in situ hybridization analysis of HER2 amplification using the CEP-17 centromere control probe (ratio of HER2/CEP-17 < 2.2).

### Preparation of single-nucleus suspensions

Nuclei from frozen tumors were isolated using a NST/DAPI buffer (800 mL of NST (146 mM NaCl, 10 mM Tris base at pH 7.8, 1 mM CaCl_2_, 21 mM MgCl_2_, 0.05% BSA, 0.2% Nonidet P-40)), 200 mL of 106 mM MgCl_2_, 10 mg of DAPI, and 5 mM EDTA. The frozen tumors were dissociated into nuclear suspensions by mincing with no.11 surgical scalpels in 1 mL of NST-DAPI cytoplasmic lysis buffer at 4C using ice blocks in a plastic Petri dish. Nuclear suspensions were filtered through 37-μm plastic mesh (Falcon). The final suspension was diluted to 1000 μL of 1 cell/50 nL with 1× PBS and D-RNase free water (0.35 × PBS in the final dilution). For the SKBR3 cell line, a 10 cm cell plate at ~100% confluence was tryspinized and washed two times with 1× PBS. The nuclei were released and stained with DAPI/NST lysis buffer, re-pelleted, suspended in DAPI-NST, and then filtered through a mesh filter. As with the tumor single-nucleus suspension, the final suspension was diluted to 1000 μL of 1 cell/50 nL with 1× PBS and D-RNase free water.

### Preparation of single cell suspension

The single cell SK-BR-3 experiment was completed on the same day as the single-nucleus SK-BR-3 experiment and used the same passage of cells. A 50% confluent 10 cm plate of SKBR3 cells was trypsinized, washed two times with 1× PBS, filtered, and stained with Hoechst 33342 and PI to distinguish live/dead cells according to the manufacturer’s recommendations (ThermoFisher). The whole-cell suspension was dispensed in the same manner as the nuclear suspension.

### Imaging QC of nuclear and cellular suspensions

To ensure that the nuclear fractions did not have any evidence of cytoplasmic membranes, we stained the nuclear fractions with Hoechst and Cytotracker Red for imaging at ×40 on the Nikon Eclipse fluorescent microscope. Similarly, we stained cellular fractions with Hoechst and Cytotracker Red to image the cytoplasm and nucleus. Z-stack images were collected in fluorescent channels, in addition to bright field imaging, and images were merged to observe the cytoplasm and nucleas of the cell suspensions.

### Nanogrid single cell/nucleus sequencing system

The ICELL8™ single cell nanogrid RNA-seq system consists of three main components. The first component is an ICELL8™ nanogrid chip manufactured in a square layout (72 × 72) in a 41 mm^2^ aluminum alloy with 5184 nanoliter wells (150 nL) using standard manufacturing processes^50^. Each nanowell was preprinted with barcoded primers (UMIs) with poly(dT) ends during manufacturing. This chip-based technology has been published previously in targeted sequencing and real-time PCR applications^51, 52^. The second component is a multisample nanodispenser that uses microsolenoid-control to precisely dispense 50 nL volumes into the nanowells. The third component is an automated imaging system composed of an Olympus BX43 microscope fitted with a ×4 objective, a robotic stage, and a CCD camera that is programmed to take images of all 5184 wells using a customized version of µManager open source software, followed by automated image analysis software called CellSelect™ that is used to analyze acquired images and identify single cell or nuclei containing wells. A more detailed technical description of the ICELL8 nanogrid single cell sequencing system and its hardware components is provided elsewhere^41^.

### Nanodispensing of nuclei and cells

Disaggregated nuclear or cellular suspensions were diluted to 20 cells/µL in eight wells of a 384-well plate (A1 through D2) and dispensed into the WaferGen ICELL8™ chip resulting in a Poisson distribution with about 30% of nanowells with single nuclei or cells. Poisson distribution of cells or nuclei in the chip nanowells is achieved because each 50 nL dispense on average dispenses a single cell when cells are at 20 cells/µL. Addressing every well of the ICELL8 chip takes 12–15 min. Every nanowell contains an adapter sequence with a well Barcode (WB, 11 nt), UMI^53^ (10 nt) and a 30-mer oligo-dT that is subsequently incorporated into the 3′ end of the transcript during WTA using the SMRT-Seq2^54^ chemistry.

### Nanowell imaging and selection of single nuclei and cells

Following cell dispensing, the microchip is centrifuged at 300×*g* for 5 min to collect cells in a single plane and the nanogrid wells are automatically imaged using an Olympus BX43 fluorescent microscope with a robotic stage. The image acquisition takes about 6 min (3 min/fluorphore). After imaging, the microchip is sealed, placed in freezing chambers, and stored at −80 °C until reverse transcription (RT). Custom CellSelect^TM^ software identifies wells with single cells, and filters cells with no cells or multiple cells based on multiple automatic and user adjustable imaging parameters. The nanowells with single cells or nuclei are then prioritized and the user can manually review images and fluorescent channels to identify live cells or nuclei for selection. A file containing positional information on identified candidate wells (dispense file) instructs the nanodispensor to deposit reagents only in the selected wells for WTA.

### 3′ RT and PCR amplification

Frozen chips were thawed, and 50 nL of RT solution (88 μL 5 × RT buffer, 44 μL 10 mM RT dNTPs, 4.4 μL 100 μM RT-E5OLIGO, 57.2 μL D-RNase-free water, and 26.4 μL 200 U/μL RT enzyme) was deposited into each selected well using the nanodepositing system. For chips with single nuclei, the 57.2 μL D-RNase free water was replaced with 52.8 μL D-RNase free water and 4.4 μL Triton X-100 to promote lysis of the cellular membrane. After RT, cDNA products from selected wells were pooled together, purified, and underwent exonuclease I treatment (2 μL 10× exonuclease buffer, 1 μL 20 U/μL exonuclease I) to remove excess, unannealed primers. The pooled barcoded cDNA libraries then underwent PCR amplification (5 μL 10× amplification buffer, 1 μL 50× amplification dNTPs, 1 μL amplification primer, 1 μL amplification enzyme, 22 μL D-RNAse free water) for 18 cycles for cells or 19 cycles for nuclei. The PCR products were purified with 0.6× AMPure XP beads and eluted in 12 μL D-RNase free water. The size distribution of cDNA was QCed using the Qubit dsDNA HS fluorometric assay and Agilent’s high sensitivity DNA chip on the Bioanalyzer system.

### Library construction and next-generation sequencing

The pooled cDNA was diluted to 0.2 ng/μL and used to construct Nextera XT (Illumina) DNA libraries with i7 index primers following the manufacturer’s instructions. The final libraries, containing barcoded single nuclei or single cell transcriptomes, were sequenced at 100 paired-end cycles on the HiSeq4000 system (Illumina). Data were processed using the CASAVA 1.8.1 pipeline (Illumina Inc.), and sequence reads were converted to a master FASTQ files.

### Bulk RNA-seq of nuclei and cells

RNA was extracted from the nuclear and cell fractions isolated from four breast cancer cell lines (MDA-MB-436, BT-549, MDA-MB-231, and T47D). One 10 cm dish at 60–85% confluence was used for each biological replicate (three biological replicates/cell line) and was washed with 9 mL of 1× PBS (−/− for calcium and magnesium). Cells were then resuspended in 1 mL trypsin and 9 mL cold PBS (1×, −/−). 60 μL of the trypsinized cells were set aside to prepare slides for whole-cell imaging and microscopy. The remainder of the cells was spun at 200 rcf for 5 min. The supernatant was subsequently removed, and the cells were resuspended again in 2 mL PBS (1×, −/−). One mL of the PBS cellular suspension was removed for whole-cell RNA extraction, spun at 100 rcf for 5 min at 4C, and resuspended in 2 mL trizol. A 20G needle was used to break apart the insoluble trizol pellets, and whole-cell RNA extraction was performed according to the manufacturer’s instructions (Fisher TR 118–100ML). The other mL of the PBS cellular suspension was removed for nuclear RNA extraction, spun at 100 rcf for 5 min at 4 °C, and resuspended in 1 mL DAPI/NST with EDTA. The DAPI/NST solution was incubated at room temperature for 4 min, spun at 8000 rcf for 5 min at 4C, and resuspended in 1 mL DAPI/NST. After a final spin at 8000 rcf for 5 min at 4C, the nuclei were resuspended in 1 mL trizol. A 20G needle was used to break apart the nuclear pellet, and an additional 1 mL of trizol was added to the suspension. Nuclear RNA extraction was performed according to the manufacturer’s instructions (Fisher TR 118–100ML).

### RNA-seq QC and data processing

The master FASTQ file containing total reads was demultiplexed into individual fastq files with each representing one single cell or one population of cells using a Perl script. Sequencing reads in each single fastq file were mapped to the human transcriptome using bowtie2^55^, and gene expression levels were summarized into expected count and transcripts per kilobase million (TPM) values using RNA-seq by expectation maximization (RSEM)^56^. Only uniquely mapped reads were used for analysis. A quality-control step was performed on the tophat2^57^ aligned bam files using RSeQC^58^ and samtools (0.1.19)^59^ to summarize distributions of reads that were mapped to rRNAs, mtRNA, introns, CDS, 5′ UTR, 3′ UTR and 10 kb up-/downstream of transcripts. The number of UMIs for each gene was counted by dropping reads that had duplicated UMIs using custom Perl scripts. However, we did not include UMI assays in this study.

### Differential gene expression analysis

We compared the gene expression in single nuclei to whole cells using a Bayesian method for SCDE^14^ analysis, which fits individual error models for single cell RNA-seq data using a Bayesian approach based on a zero-inflated negative binomial model process. The differentially expressed genes are defined as FDR-adjusted *p* value <0.05 and |log_2_(fold change)|> = 1. We removed genes that were detected with counts <10 in <30 cells. We applied an Empirical Bayes hierarchical model (EBSeq)^60^ for population RNA-seq differential expression analysis at gene levels. The differentially expressed genes are defined as those had posterior probability of being DE >0.95 and |log_2_(fold change)|> = 1. Clustered heat maps of gene expression were generated with R package “heatmap3”^20^ based on log_2_(count + 1), log_2_(TPM + 1), or z-scores. Differentially expressed genes were analyzed with Ingenuity IPA for pathway analysis and cellular organelle localization annotations. To identify differentially expressed cancer genes in TNBC tumor cells, we combined the three subpopulations of single nuclei that had aneuploidy copy number aberrations (CNAs) profiles as tumor cell populations and combined the predicted matched normal nuclei and normal nuclei from another patient as the normal cell populations. We then performed differential gene expression analysis between the two groups of single nuclei using MAST^35, 36^. The differential genes were defined as having FDR-adjusted *p* value <0.05 and |log_2_ (fold change)| ≥ 1. Finally, differentially expressed cancer genes in tumor cells were identified by intersecting the DE gene list with T200 clinical gene panel and with a 40-gene TCGA breast cancer gene list.

### Breast cancer subtype prediction

We used the intrinsic gene centroids signature (PAM50)^61^ to classify single cells and nuclei into five established intrinsic breast cancer molecular subtypes (normal-like, basal, luminal A, luminal B, and Her2 amplification) using “genefu” package^62^ with log_2_(TPM + 1) data matrix. Single cells or nuclei with low prediction confidence (<0.7) are set as undefined. In a separate analysis, single nuclei were classified into six TNBC subtypes, including two basal-like (BL1 and BL2), an IM, a mesenchymal (M), a MSL, and a LAR subtype^63^. The ER+ cells were excluded from the TNBC subtype prediction.

### GSVA

We applied single-sample GSVA (ssGSVA)^31^ to determine the molecular phenotypes of single cells and nuclei using log_2_(TPM + 1) data. We first obtained GSVA scores for 189 oncogenic gene sets (MSiDB version 5.2)^32^ for each single nuclei or whole-cell sample, and then compared the nuclei enrichment scores to whole cells by using R package “limma”^64^. Differentially enriched gene sets were defined as FDR-adjusted *p* value <0.05 and |score difference|> = 0.1.

### Cell cycle analysis of single nuclei

Cell cycle genes from gene ontology set (version 5.2 MSiDB)^65–67^ with annotations of “G1 phase of mitotic cell cycle” for G1 phase genes, “S phase of mitotic cell cycle” for S phase genes, “M phase of mitotic cell cycle” for M phase genes were used. A G2 phase gene list that was previously defined in synchronized HeLa cells was also used^68^. We then defined the four cell phase (G1, S, G2, and M) scores as the average expression [log_2_(TPM + 1)] of curated cell cycle genes and defined the cell cycle phase by hierarchical clustering of centered phase scores using R package “heatmap3”^20^.

### High-dimensional reduction data analysis

The normalized log_2_(count + 1) matrix was centered and scaled to z-scores to perform PCA using “prcomp” function in R (www.r-project.org). Genes that were detected in <30 cells were excluded from the analysis. The first five components were selected based on “elbow” principle and top 20 loading genes were sent for clustering using a SNN modularity optimization-based method^37^ and then marker genes for each detected clusters were selected using normalized log_2_(count + 1) matrix with the “bimodal”-based gene expression analysis^69^ that was built in the “FindAllMarkers” function in R package “Seurat”^70^. The t-SNE^38^ method was applied based on the pair-wise Euclidean distances of the log_2_(count + 1) matrix. Hierarchical clustering with “ward.D2” linkage and “Euclidean” distance was performed on log_2_(count + 1) matrix or z-scores using R package “heatmap3”^20^.

### Copy number calculation from single cell RNA data

Single cell and nuclei copy number was calculated from the log_2_(TPM + 1) matrix using a “moving average” approach that was adapted from a previous study^3^. We use the log_2_(TPM + 1) values as gene expression values and we further scaled the total expression of all cells to 100,000 to normalize gene expressions within each single cells to comparable scales and avoid floating the variance among highly expressed genes. We sorted the analyzed genes by their genomic coordinates that were annotated by University of California Santa Cruz gene list containing a total of 23,346 genes. We excluded genes that have expression values <0.15 on average, and ended up with ~3000 genes across the genome and ~130 genes/chromosome on average (except that Y chromosome had only one or two genes). To define the copy number baseline, we also sequenced a set of 380 normal breast tissue single cells, and took their average expressions of each gene as the normal copy number base line. We normalized single cell gene expression to this baseline to obtain a relative gene expression for each gene location. To mitigate the bias caused by extreme gene expression levels, we replaced the relative gene expression values that are >3 with 3 and relative expressions <−3 with −3. We then obtained “moving average” of adjacent 50 gene relative expression values to represent the log_2_(copy number ratio) of the genomic location. We normalized the log_2_(copy number ratio) to their mean values for each cell to center around zeros. Last, we performed hierarchical clustering^20^ of all tested single cell CNAs with the normal breast tissue cells to identify aneuploid tumor and normal diploid cell populations.

### Data availability

The data from this study have been deposited into the Sequencing Read Archive and are available for download under accession SRP095350.

## Electronic supplementary material

Supplementary Information
