# Supplementary material for: Nanogrid single-nucleus RNA sequencing reveals phenotypic diversity in breast cancer
Source: Nat Commun. 2017 Aug 9;8:228. doi: 10.1038/s41467-017-00244-w (PMC5550415; doi:10.1038/s41467-017-00244-w)
Supplement: Supplementary file 1 — Supplementary Information [file 41467_2017_244_MOESM1_ESM.pdf]

File name: Supplementary Information
Description: Supplementary figures and supplementary tables.

Supplementary Figures

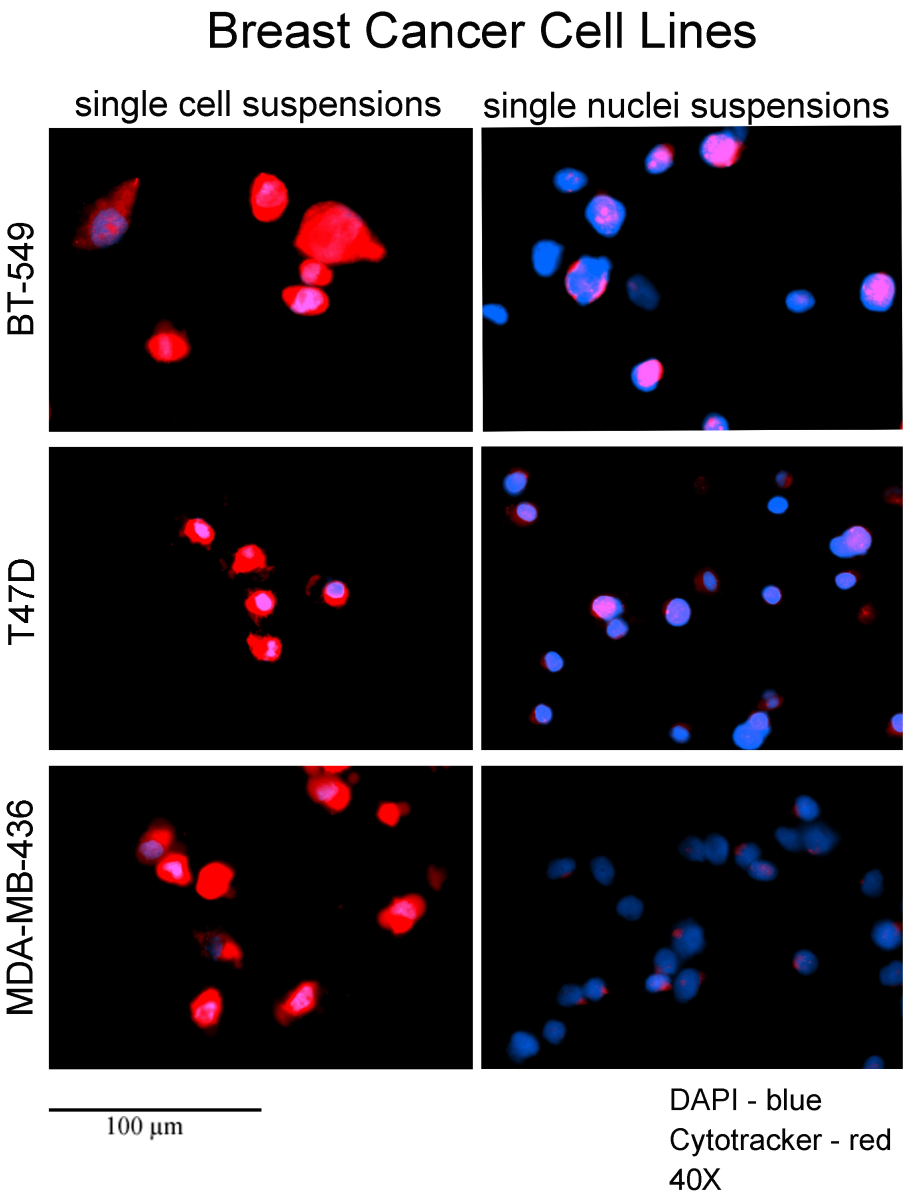

Supplementary Figure 1 - Imaging of Single Cell and Nuclei Suspensions. Purified suspensions of single cells and single nuclei from the MDA-MB436 breast cancer cell line were stained with DAPI (nuclei) or Hoechst (cells) and imaged using fluorescence and brightfield microscopy at 40X magnification. Although the majority of DAPI is excluded by the cell membrane in living cells, there is always a small amount that permeates and shows low-level staining of the nucleus of live cells. However these levels are much lower compared to the DAPI staining in the isolated nuclei (right panels).

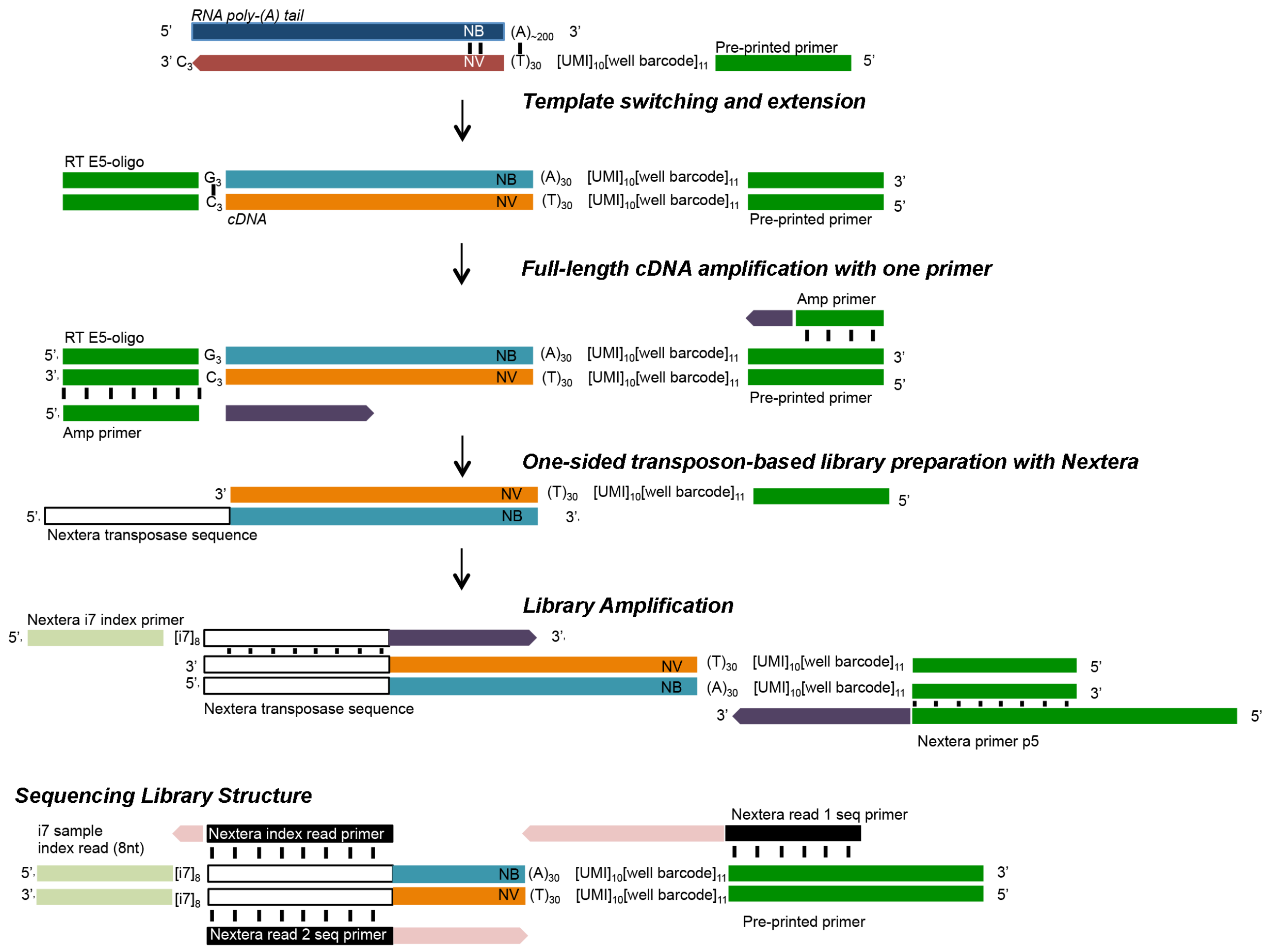

Supplementary Figure 2 – Construction of Nanowell Barcoded Single Cell 3' RNA Sequencing Libraries

3' Single cell RNA sequencing is performed by hybridization of the polyA mRNA tail to an oligo dT_{30} adapter with a 11bp well barcode and 10bp UMI that is pre-printed in each nanowell. Single cells or nuclei undergo reverse-transcription using template switching and extension, followed by full-length cDNA amplification. A one-sided tagmentation reaction is then used to add a Nextera transposase adapter to the 5' end (the other strand is omitted for illustrative purposes). PCR is performed using the nextera P5 primer and i7 index primer to extend the adapter sequences and enrich the library concentrations. The final sequencing reaction involves 3 sequencing primers to read the WBC and UMI (read1), the 3' RNA sequence (read 2) and the i7 nextera sample index (read3). In this figure N represents A, C, G, or T; B represents C, G, or T; and V represents A, C, or G. Dotted lines indicate where the first nucleotide hybridizations occur.

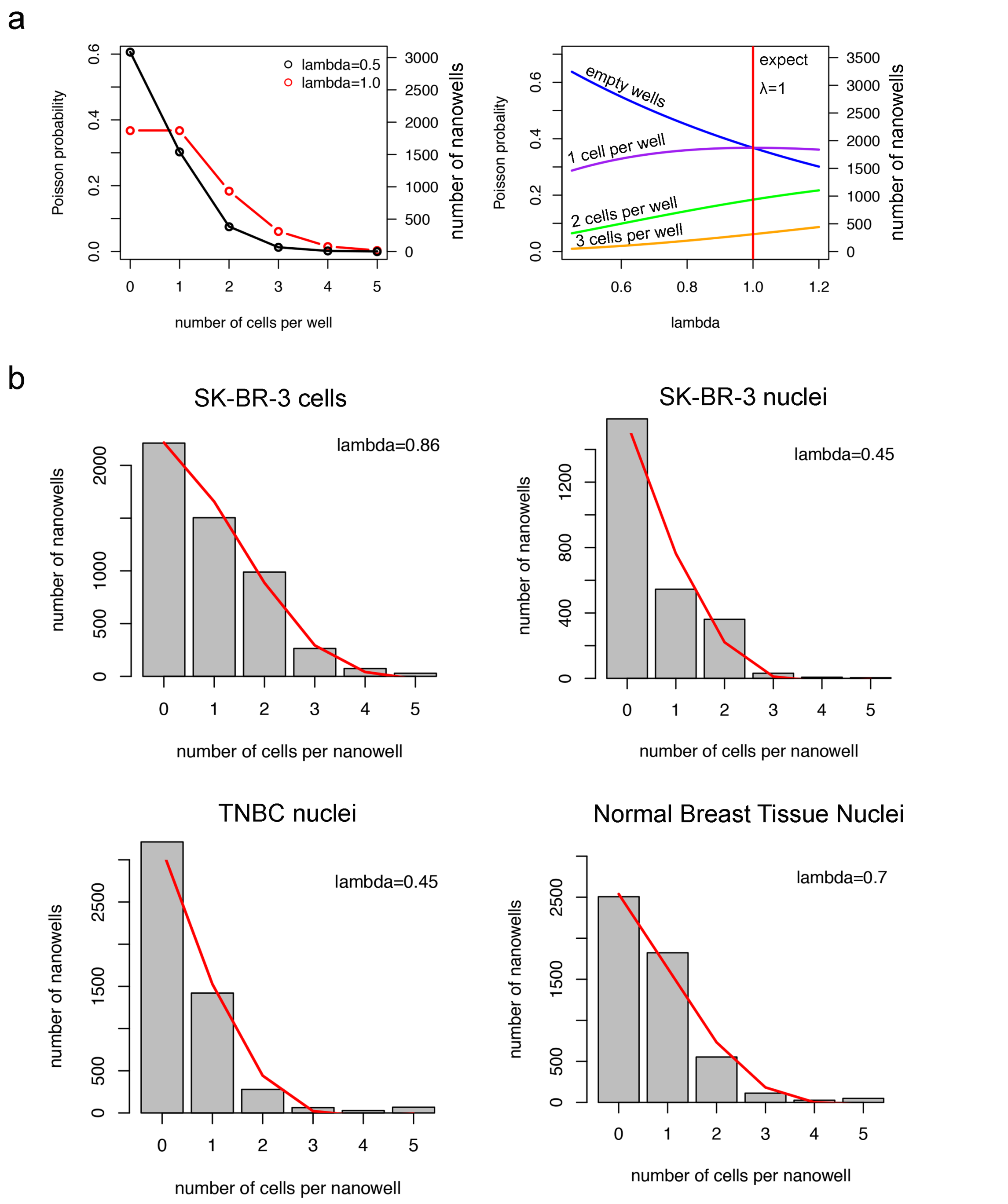

Supplementary Figure 3 –Poisson Distributions of Cells and Nuclei After Nanodispensing

(a) Theoretical Poisson distribution for the number of expected single cells per nanowell (left panel) and probability distribution under series of number of cells per well with expected lambda=1, where ~1800 wells with single cells/nuclei are expected to be selected for analysis per chip (right panel). (b) Experimental distributions of cell or nuclei numbers deposited into nanowells from SK-BR-3 cell suspensions, SK-BR-3 nuclear suspensions, TNBC nuclear suspensions and a normal breast tissue nuclei suspension, with observed lambda range from 0.45-0.86.

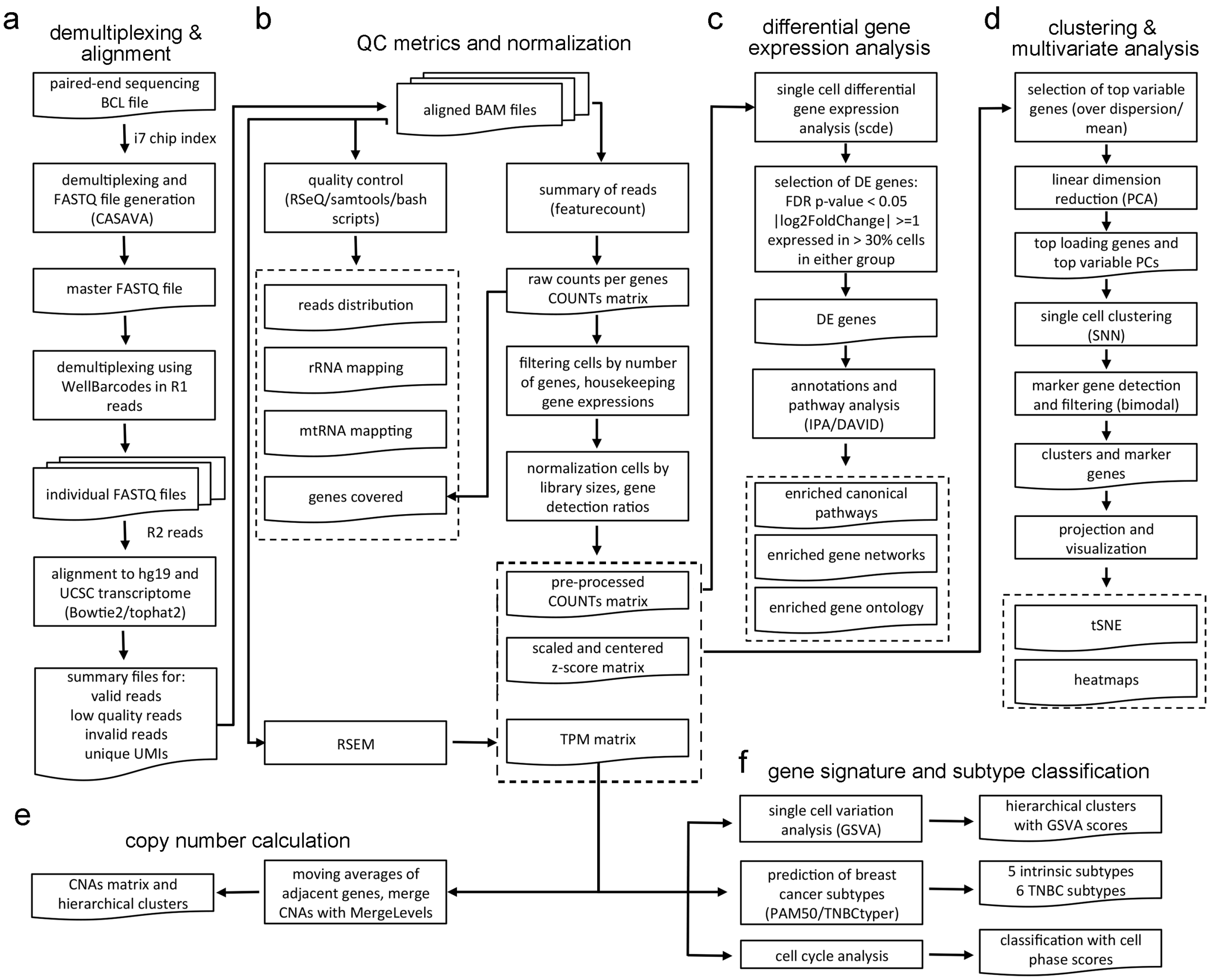

Supplementary Figure 4 – Single Nuclei and Cell RNA Data Analysis Workflow
Data processing and analysis workflow for single nuclei and single cell RNA data. (a) Sequence reads are demultiplexed by well barcodes (WBC) and aligned to the human genome reference assembly. (b) Quality control metrics are calculated and read counts are normalized by library size and housekeeping genes, transformed to z-scores or TPM using RSEM. (c) Differentially expressed genes are identified using SCDE and annotated for gene ontologies and pathways. (d) Clustering and multivariate analysis is performed to identify groups of cells with similar transcriptional profiles. (e) Copy number profiles are calculated from RNA profiles. (f) Gene signature analysis and classification of breast cancer subtypes is performed.

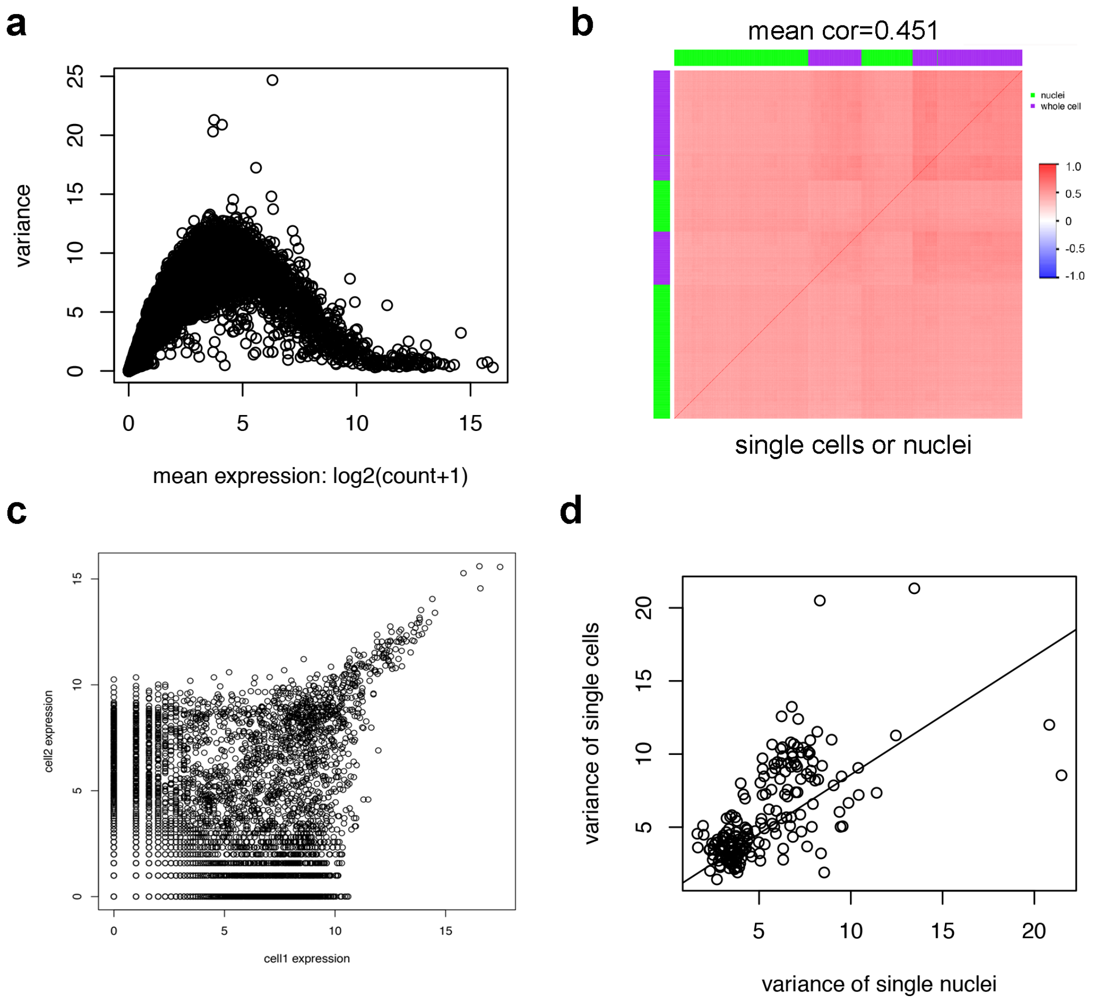

Supplementary Figure 5 – Variance in Single Cell Gene Expression Data
Calculation of data variation between gene expression levels in single cell and single nuclei data from SK-BR-3. (a) Variance of all detectable genes in SK-BR-3 single cell data as a function of mean expression levels. (b) Pair-wise correlation heatmap of all single nuclei and single cells using 4414 top detected genes. (c) Scatter plot of two representative single cell gene expression levels in log2(count+1) scale. (d) Scatter plot of single cell and single nuclei within group variations of 196 significant genes detected between both groups by SCDE.

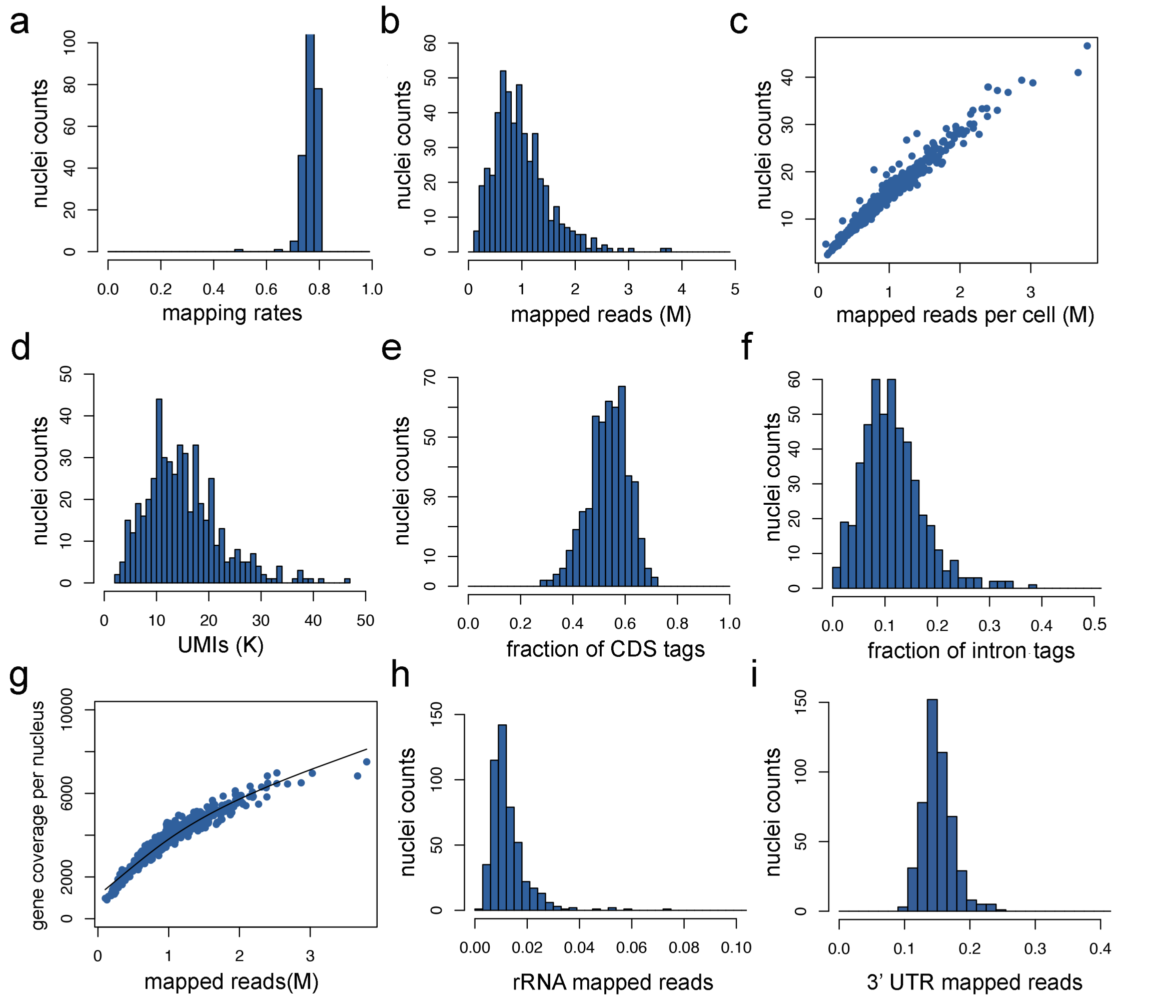

Supplementary Figure 6 - QC Metrics for the Single Breast Tumor Nuclei
Sequence data metrics were calculated for single nuclei from the triple-negative breast tumor analyzed by nanogrid SNRS, including: (a) Sequence read mapping rates to the human genome reference assembly, (b) number of mapped reads per nucleus, (c) number of mapped reads in millions, (d) number of unique molecular identifiers detected in single nuclei, (e) read tags mapped to coding regions, (f) read tags mapped to intronic regions, (g) number of mapped reads per cell as a function of gene coverage, (h) ribosomal RNA mapped reads, (i) mapped reads to the 3' untranslated region.

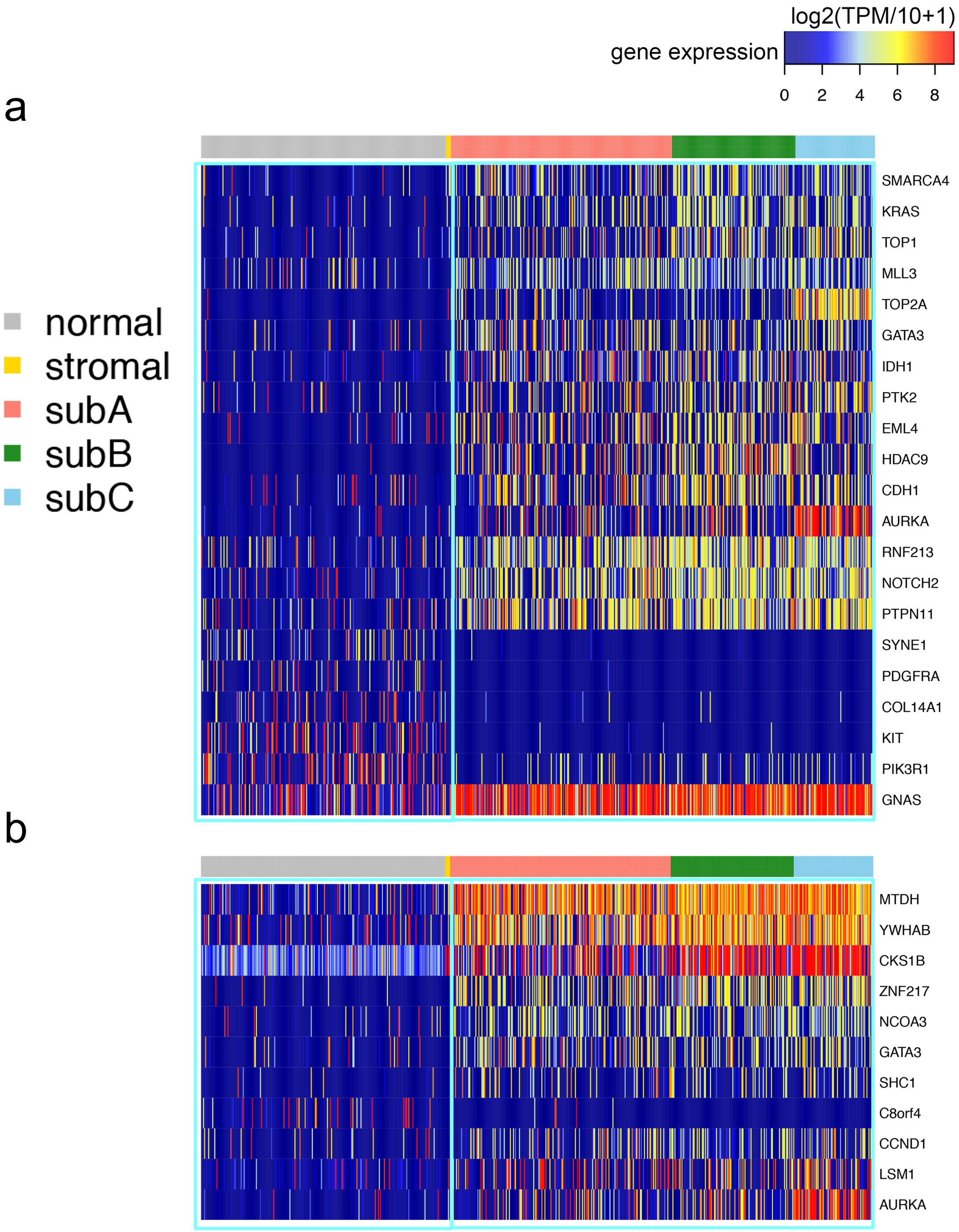

Supplementary Figure 7 –Expression of Cancer Genes in Single Cells from Stromal and Tumor Subpopulations

Expression of known cancer genes in single cells from normal breast tissues, stroma and tumor tissues using targeted breast cancer gene sets from (a) TCGA and (b) the T200 cancer gene panel. Subpopulations are annotated based on detection of differential gene expression of all genes in Figure 5c and 5d.

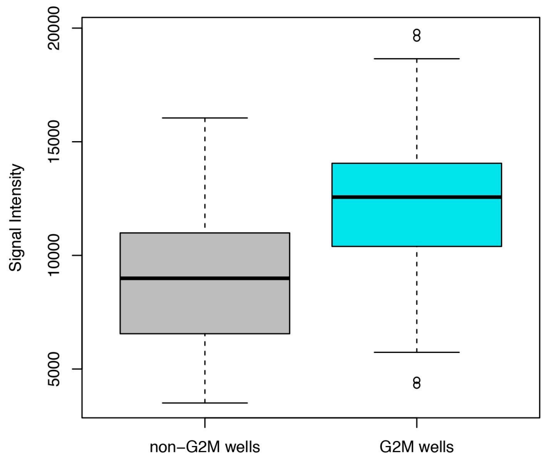

Supplementary Figure 8 – Comparison of G2/M single nuclei sizes from nanogrid imaging data
Boxplots of DAPI-staining intensities of G2/M single nuclei and G1/0/S single nuclei as inferred by RNA-seq data was matched to automated imaging data from the nanogrid system, showing larger nuclear sizes in the G2/M cells. The boxes indicated the 75% IQR and whiskers showed the default values of the boxplot (*graphics*). N= 78 for G2M group and N=338 for the non-G2M group.

Supplementary Tables

Supplementary Table 1 - Upregulated Genes in SK-BR-3 Single Nuclei comparing to Cells

This table lists all of the significant upregulated genes in nuclei compared to cells. Columns listed include Gene Identifier (Gene ID), Fold-change, Entrez Gene Name, cellular localization and gene class.

Gene ID	FoldChange	Entrez Gene Name	cellular localization	gene class
ANKRD20A9P	2.0	ankyrin repeat domain 20 family member A9, pseudogene	Other	other
ARHGEF26-AS1	2.8	ARHGEF26 antisense RNA 1	Other	other
ATP6V0A2	2.8	ATPase H+ transporting V0 subunit a2	Cytoplasm	transporter
BRIP1	2.1	BRCA1 interacting protein C-terminal helicase 1	Nucleus	enzyme
C12orf65	2.2	chromosome 12 open reading frame 65	Other	other
C21orf62	2.8	chromosome 21 open reading frame 62	Other	other
CA5B	2.6	carbonic anhydrase 5B	Cytoplasm	enzyme
CBFA2T2	2.7	CBFA2/RUNX1 translocation partner 2	Nucleus	transcription regulator
CCDC142	2.2	coiled-coil domain containing 142	Other	other
CEACAM22P	3.2	carcinoembryonic antigen related cell adhesion molecule 22, pseudogene	Other	other
CEP104	2.1	centrosomal protein 104	Cytoplasm	other
CMBL	2.4	carboxymethylenebutenolidase homolog (Pseudomonas)	Cytoplasm	enzyme
CNNM3	2.6	cyclin and CBS domain divalent metal cation transport mediator 3	Other	other
CRX	2.1	cone-rod homeobox	Nucleus	transcription regulator
CYP4V2	2.8	cytochrome P450 family 4 subfamily V member 2	Cytoplasm	enzyme
DCAF10	2.0	DDB1 and CUL4 associated factor 10	Other	other
DCUN1D2	2.0	defective in cullin neddylation 1 domain containing 2	Other	other
FAM116A	2.2	DENN domain containing 6A	Cytoplasm	other
DFFB	2.3	DNA fragmentation factor subunit beta	Nucleus	enzyme
DMC1	3.2	DNA meiotic recombinase 1	Nucleus	enzyme
ENTPD4	2.4	ectonucleoside triphosphate diphosphohydrolase 4	Cytoplasm	enzyme
EPHA10	3.1	EPH receptor A10	Plasma Membrane	transmembrane receptor
ERVV-1	2.8	endogenous retrovirus group V member 1	Other	other
EXD1	2.7	exonuclease 3'-5' domain containing 1	Other	other
FAM126B	2.5	family with sequence similarity 126 member B	Other	other
FAM74A1	2.9	family with sequence similarity 74 member A1	Other	other
FBLIM1	2.1	filamin binding LIM protein 1	Plasma Membrane	other
FBXO27	2.4	F-box protein 27	Other	other
FLJ42102	2.5	uncharacterized LOC399923	Other	other
FMN1	2.1	formin 1	Nucleus	other
GFOD2	3.0	glucose-fructose oxidoreductase domain containing 2	Extracellular Space	enzyme
GGA2	2.0	golgi associated, gamma adaptin ear containing, ARF binding protein 2	Cytoplasm	transporter
GNE	2.5	glucosamine (UDP-N-acetyl)-2-epimerase/N-acetylmannosamine kinase	Cytoplasm	kinase
GREB1	2.4	growth regulation by estrogen in breast cancer 1	Cytoplasm	other
GRIPAP1	2.3	GRIP1 associated protein 1	Plasma Membrane	other
HNRNPA1P10	3.1	heterogeneous nuclear ribonucleoprotein A1 pseudogene 10	Other	other
IGF2R	2.1	insulin like growth factor 2 receptor	Plasma Membrane	transmembrane receptor
IL17RD	2.4	interleukin 17 receptor D	Cytoplasm	other
IRF1	2.5	interferon regulatory factor 1	Nucleus	transcription regulator
KBTBD12	2.9	kelch repeat and BTB domain containing 12	Other	other
KDELC2	2.0	KDEL motif containing 2	Other	other
L1TD1	3.3	LINE-1 type transposase domain containing 1	Other	other
LAIR1	3.0	leukocyte associated immunoglobulin like receptor 1	Plasma Membrane	transmembrane receptor
LINC00294	2.2	long intergenic non-protein coding RNA 294	Other	other
LINC00410	5.1	long intergenic non-protein coding RNA 410	Other	other
C14orf23	2.6	long intergenic non-protein coding RNA 1551	Other	other
LOC100190986	4.0	uncharacterized LOC100190986	Other	other
LOC643406	2.4	uncharacterized LOC643406	Other	other
LYZ	2.6	lysozyme	Extracellular Space	enzyme
MAB21L3	2.9	mab-21 like 3	Other	other
MAP1LC3C	2.2	microtubule associated protein 1 light chain 3 gamma	Cytoplasm	other
MAP7D3	2.3	MAP7 domain containing 3	Cytoplasm	other
MBOAT1	2.4	membrane bound O-acyltransferase domain containing 1	Cytoplasm	other
MEFV	3.1	Mediterranean fever	Nucleus	other
MTFMT	2.1	mitochondrial methionyl-tRNA formyltransferase	Cytoplasm	enzyme
LOC90834	3.9	NA	NA	NA
LOC731424	3.4	NA	NA	NA
LOC728558	3.0	NA	NA	NA
LOC147670	3.0	NA	NA	NA
LOC145663	2.5	NA	NA	NA
LOC100190938	2.5	NA	NA	NA
LOC642236	2.4	NA	NA	NA
NLRP12	2.9	NLR family, pyrin domain containing 12	Cytoplasm	other
NPIPL3	3.4	nuclear pore complex interacting protein family member B3	Other	other
NT5DC3	2.5	5'-nucleotidase domain containing 3	Other	other
NUAK2	2.9	NUAK family kinase 2	Other	kinase
NXN	2.4	nucleoredoxin	Nucleus	enzyme
ORAI2	2.6	ORAI calcium release-activated calcium modulator 2	Plasma Membrane	other

OTUD6A	2.1	OTU deubiquitinase 6A	Other	peptidase
P2RX5-TAX1BP3	2.4	P2RX5-TAX1BP3 readthrough (NMD candidate)	Other	other
PDDC1	2.1	Parkinson disease 7 domain containing 1	Cytoplasm	other
PDE6A	2.4	phosphodiesterase 6A	Plasma Membrane	enzyme
PGM2L1	2.0	phosphoglucomutase 2-like 1	Cytoplasm	enzyme
PGM5P2	2.1	phosphoglucomutase 5 pseudogene 2	Other	other
PPP1R3B	3.2	protein phosphatase 1 regulatory subunit 3B	Cytoplasm	other
PRR11	2.2	proline rich 11	Other	other
PSTPIP2	2.2	proline-serine-threonine phosphatase interacting protein 2	Cytoplasm	other
RBMS2	2.2	RNA binding motif single stranded interacting protein 2	Nucleus	other
RPS6KA3	2.3	ribosomal protein S6 kinase A3	Cytoplasm	kinase
RRP7B	3.0	ribosomal RNA processing 7 homolog B, pseudogene	Other	other
KIAA0226	2.0	RUN and cysteine rich domain containing beclin 1 interacting protein	Cytoplasm	other
RUNDC1	2.2	RUN domain containing 1	Other	other
SCAI	2.3	suppressor of cancer cell invasion	Nucleus	transcription regulator
SCD5	3.4	stearoyl-CoA desaturase 5	Cytoplasm	enzyme
SERPINB9	2.3	serpin family B member 9	Cytoplasm	other
SGSM1	3.3	small G protein signaling modulator 1	Nucleus	other
SKA1	2.1	spindle and kinetochore associated complex subunit 1	Nucleus	other
MCART1	2.3	solute carrier family 25 member 51	Cytoplasm	other
SLC7A5P2	2.9	solute carrier family 7 member 5 pseudogene 2	Other	other
SPRED1	2.5	sprouty related EVH1 domain containing 1	Plasma Membrane	other
STAC2	3.1	SH3 and cysteine rich domain 2	Other	other
FAM40B	2.4	striatin interacting protein 2	Cytoplasm	other
STYX	2.1	serine/threonine/tyrosine interacting protein	Cytoplasm	phosphatase
TACC2	3.2	transforming acidic coiled-coil containing protein 2	Nucleus	other
TADA2B	2.5	transcriptional adaptor 2B	Nucleus	other
TAF8	2.0	TATA-box binding protein associated factor 8	Nucleus	transcription regulator
TBXA2R	2.6	thromboxane A2 receptor	Plasma Membrane	G-protein coupled receptor
TLCD2	2.6	TLC domain containing 2	Other	other
TNFAIP8L1	2.9	TNF alpha induced protein 8 like 1	Cytoplasm	other
TSIX	2.6	TSIX transcript, XIST antisense RNA	Nucleus	other
UCKL1-AS1	3.2	UCKL1 antisense RNA 1	Other	other
UTP3	2.2	UTP3, small subunit processome component homolog (S. cerevisiae)	Nucleus	other
VPS53	2.2	VPS53, GARP complex subunit	Cytoplasm	other
VSIG1	3.2	V-set and immunoglobulin domain containing 1	Plasma Membrane	other
VSTM4	3.3	V-set and transmembrane domain containing 4	Other	other
WDR45	2.2	WD repeat domain 45	Other	other
WNT7B	2.4	Wnt family member 7B	Extracellular Space	other
XIST	2.1	X inactive specific transcript (non-protein coding)	Nucleus	other
ZC3H12D	3.1	zinc finger CCCH-type containing 12D	Cytoplasm	other
ZFYVE19	2.6	zinc finger FYVE-type containing 19	Cytoplasm	other
ZKSCAN3	2.6	zinc finger with KRAB and SCAN domains 3	Nucleus	transcription regulator
ZMAT3	2.3	zinc finger matrin-type 3	Nucleus	other
ZNF124	2.3	zinc finger protein 124	Nucleus	other
ZNF264	2.6	zinc finger protein 264	Nucleus	other
ZNF347	2.5	zinc finger protein 347	Nucleus	other
ZNF490	2.5	zinc finger protein 490	Nucleus	other
ZNF526	2.2	zinc finger protein 526	Other	other
ZNF793	2.6	zinc finger protein 793	Other	other
ZNF805	2.8	zinc finger protein 805	Other	other
ZNF814	2.7	zinc finger protein 814	Other	other
ZSCAN22	2.7	zinc finger and SCAN domain containing 22	Nucleus	transcription regulator

Supplementary Table 2 - Downregulated Genes in SK-BR-3 Single Nuclei comparing to Cells

This table lists all of the significant downregulated genes in nuclei compared to cells. Columns listed include Gene Identifier (Gene ID), Fold-change, Entrez Gene Name, cellular localization and gene class.

Gene ID	FoldChange	Entrez Gene Name	cellular localization	gene class
PTS	-2.3	6-pyruvoyltetrahydropterin synthase	Cytoplasm	enzyme
AP2S1	-2.2	adaptor related protein complex 2 sigma 1 subunit	Cytoplasm	transporter
ARF4	-2.1	ADP ribosylation factor 4	Cytoplasm	enzyme
ALG14	-2.5	ALG14, UDP-N-acetylglucosaminyltransferase subunit	Cytoplasm	enzyme
ASNS	-2.2	asparagine synthetase (glutamine-hydrolyzing)	Cytoplasm	enzyme
BOLA3	-2.6	bolA family member 3	Other	other
CD9	-2.3	CD9 molecule	Plasma Membrane	other
CKS1B	-2.4	CDC28 protein kinase regulatory subunit 1B	Other	kinase
CETN2	-2.1	centrin 2	Nucleus	enzyme
C11orf73	-2.0	chromosome 11 open reading frame 73	Cytoplasm	transporter
COMMD3	-2.3	COMM domain containing 3	Other	other
COPS4	-2.0	COP9 signalosome subunit 4	Cytoplasm	other
CNIH	-2.3	cornichon family AMPA receptor auxiliary protein 1	Plasma Membrane	other
C7orf44	-2.5	cytochrome c oxidase assembly factor 1 homolog	Cytoplasm	other
DAZAP2	-2.0	DAZ associated protein 2	Nucleus	other
DRAM2	-2.5	DNA damage regulated autophagy modulator 2	Cytoplasm	other
ESD	-2.3	esterase D	Cytoplasm	enzyme
EIF5A	-2.0	eukaryotic translation initiation factor 5A	Cytoplasm	translation regulator
FOS	-2.1	FBJ murine osteosarcoma viral oncogene homolog	Nucleus	transcription regulator
GABARAPL2	-2.5	GABA type A receptor associated protein like 2	Cytoplasm	other
GTF2IP1	-5.3	general transcription factor IIi pseudogene 1	Other	other
GNPDA1	-2.2	glucosamine-6-phosphate deaminase 1	Cytoplasm	enzyme
GSTM4	-2.0	glutathione S-transferase mu 4	Cytoplasm	enzyme
CGA	-2.1	glycoprotein hormones, alpha polypeptide	Extracellular Space	other
H3F3AP4	-2.7	H3 histone, family 3A, pseudogene 4	Other	other
HIGD1A	-2.1	HIG1 hypoxia inducible domain family member 1A	Cytoplasm	other
HAT1	-2.1	histone acetyltransferase 1	Nucleus	enzyme
IGFL1	-3.6	IGF like family member 1	Extracellular Space	other
ID1	-3.1	inhibitor of DNA binding 1, HLH protein	Nucleus	transcription regulator
ID3	-2.0	inhibitor of DNA binding 3, HLH protein	Nucleus	transcription regulator
ITGB3BP	-2.2	integrin subunit beta 3 binding protein	Nucleus	other
JKAMP	-2.9	JNK1/MAPK8-associated membrane protein	Cytoplasm	other
LAMTOR1	-2.0	late endosomal/lysosomal adaptor, MAPK and MTOR act	Plasma Membrane	other
MAD2L1	-2.1	MAD2 mitotic arrest deficient-like 1 (yeast)	Nucleus	other
MEMO1	-2.3	mediator of cell motility 1	Cytoplasm	other
MT1X	-2.2	metallothionein 1X	Other	other
MGST1	-2.0	microsomal glutathione S-transferase 1	Cytoplasm	enzyme
BRP44	-2.6	mitochondrial pyruvate carrier 2	Plasma Membrane	other
MRPL39	-2.1	mitochondrial ribosomal protein L39	Cytoplasm	other
MRPS14	-2.2	mitochondrial ribosomal protein S14	Cytoplasm	other
MRPS23	-2.1	mitochondrial ribosomal protein S23	Cytoplasm	other
LOC440354	-2.1	NA	NA	NA
LOC541471	-2.1	NA	NA	NA
LOC550643	-2.0	NA	NA	NA
NDUFAF4	-2.2	NADH:ubiquinone oxidoreductase complex assembly fact	Cytoplasm	other
NIT2	-2.2	nitrilase family member 2	Cytoplasm	enzyme
NUTF2	-2.0	nuclear transport factor 2	Nucleus	transporter
C20orf111	-2.2	oxidative stress responsive serine rich 1	Other	enzyme
PDZD11	-2.5	PDZ domain containing 11	Extracellular Space	other
PRDX3	-2.3	peroxiredoxin 3	Cytoplasm	enzyme
PIGU	-2.3	phosphatidylinositol glycan anchor biosynthesis class U	Cytoplasm	enzyme
PHLDA2	-2.9	pleckstrin homology like domain family A member 2	Cytoplasm	other
POLR2G	-2.0	polymerase (RNA) II subunit G	Nucleus	enzyme
POLR2K	-2.5	polymerase (RNA) II subunit K	Nucleus	enzyme
PFDN1	-2.2	prefoldin subunit 1	Cytoplasm	transcription regulator
PPP6C	-2.0	protein phosphatase 6 catalytic subunit	Nucleus	phosphatase

PLP2	-2.5	proteolipid protein 2 (colonic epithelium-enriched)	Cytoplasm	transporter
RPA3	-2.0	replication protein A3	Nucleus	other
RPL21	-2.7	ribosomal protein L21	Cytoplasm	other
SAMM50	-2.2	SAMM50 sorting and assembly machinery component	Cytoplasm	other
C19orf42	-2.2	small integral membrane protein 7	Other	other
TAX1BP3	-2.6	Tax1 binding protein 3	Cytoplasm	transcription regulator
TXNDC12	-2.3	thioredoxin domain containing 12	Cytoplasm	enzyme
TMX2	-2.2	thioredoxin related transmembrane protein 2	Other	enzyme
TMEM106C	-2.5	transmembrane protein 106C	Other	other
TMEM14A	-3.6	transmembrane protein 14A	Other	other
TMEM18	-2.3	transmembrane protein 18	Nucleus	other
TMEM99	-2.6	transmembrane protein 99	Other	other
TSFM	-2.5	Ts translation elongation factor, mitochondrial	Cytoplasm	translation regulator
UBE2T	-2.0	ubiquitin conjugating enzyme E2 T	Nucleus	enzyme
VPS25	-2.6	vacuolar protein sorting 25 homolog	Cytoplasm	other
VDAC3	-2.0	voltage dependent anion channel 3	Cytoplasm	ion channel
WDR83OS	-2.1	WD repeat domain 83 opposite strand	Other	other
WDYHV1	-2.5	WDYHV motif containing 1	Cytoplasm	other
ZNF706	-2.0	zinc finger protein 706	Other	other

Supplementary Table 3 - Differentially Enriched Oncogenic Gene Signatures Between SK-BR-3 Single Nuclei and Cells

This table lists the 5 out of 189 significant differentially enriched oncogenic gene signatures identified by GSVA analysis between single nuclei and single cells. Columns listed include the Gene Signature Identifier, Average difference of GSVA score between nuclei and whole cells (Avg_diff), p-value (pval) and FDR adjusted p-value (adjPval).

Nuclei Enriched oncogenic genesets

GeneSet Identifier	Avg_diff	pval	adjPval
CSR LATE UP.V1 UP	0.118060921	7.22E-38	1.14E-36
EIF4E UP	0.11607917	3.70E-56	2.33E-54
RB DN.V1 UP	0.102414964	1.19E-40	2.49E-39

Whole Cell Enriched oncogenic genesets

GeneSet Identifier	Avg_diff	pval	adjPval
HINATA NFKB IMMU INF	-0.10104583	1.38E-13	5.23E-13
CSR EARLY UP.V1 DN	-0.11034172	2.12E-73	4.01E-71

Supplementary Table 4 – Comparison of Sequencing Metrics of Nanogrid SNRS-seq to Drop-seq.

This table lists sequencing and QC metrics for single cells that were profiled by nanogrid SNRS and Drop-seq from the SK-BR-3 cell line. To perform a fair comparison the Nanogrid data was downsampled to 90,732 reads per cell before metrics were calculated.

Platforms	Drop-seq	Nanogrid*
median total reads per cell	90,732	90,732
number of cells sequenced	8,128	525
median mapping rates per cell	29.00%	68.72%
median mapped reads per cell	26,312	62,354
median CDS reads/tags fractions	30.60%	72.26%
median Intron reads/tags fractions	6.00%	1.33%
median gene covered per cell	2,714	2,349
median UMIs per cell	16,414	25,061

*data were downsampled from original sequenced fastq files.
